# Nanocrystal-Embedded-Insulator (NEI) Ferroelectric FETs for Negative Capacitance Device and Non-Volatile Memory Applications

**DOI:** 10.1186/s11671-019-2943-9

**Published:** 2019-04-01

**Authors:** Yue Peng, Genquan Han, Wenwu Xiao, Jibao Wu, Yan Liu, Jincheng Zhang, Yue Hao

**Affiliations:** 1 State Key Discipline Laboratory of Wide Band Gap Semiconductor Technology, School of Microelectronics, Xidian University, Xi’an, 710071 China; 2 School of Materials Science and Engineering, Xiangtan University, Xiangtan, 411105 China

**Keywords:** NEI, Ferroelectric, NC, Memory, Germanium, FeFET

## Abstract

We report a novel nanocrystal-embedded-insulator (NEI) ferroelectric field-effect transistor (FeFET) with very thin unified-ferroelectric/dielectric (FE/DE) insulating layer, which is promising for low-voltage logic and non-volatile memory (NVM) applications. The ferroelectric nature of the NEI layers comprising orthorhombic ZrO_2_ nanocrystals embedded in amorphous Al_2_O_3_ is proved by polarization voltage measurements, piezoresponse force microscopy, and electrical measurements. The temperature dependent performance and endurance behavior of a NEI negative capacitance FET (NCFET) are investigated. A FeFET with 3.6 nm thick FE/DE achieves a memory window larger than 1 V, paving a pathway for ultimate scaling of FE thickness to enable three-dimensional FeFETs with very small fin pitch.

## Background

**Fig. 1:**
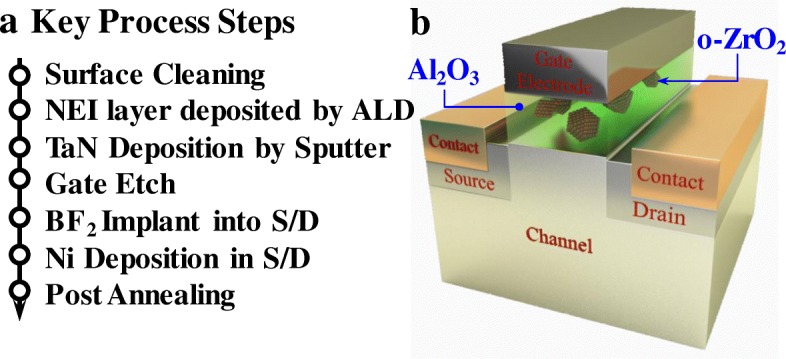
**a** Key process steps for the fabrication of the NEI ferroelectric field-effect transistors. **b** 3D schematic of the fabricated NEI FeFET

Field-effect transistors with a ferroelectric gate insulator layer (FeFETs) have attracted considerable interest for a variety of integrated circuit applications. Due to its inherent negative capacitance (NC) properties, a FeFET can achieve steeper switching behavior than a conventional MOSFET, enabling lower voltage operation [1]. Various channel structures [2–4] and materials [5–7] have obtained sub-60 mV/decade subthreshold swing (SS). Also, hysteresis in the current-voltage (*I*-*V*) characteristic due to remnant polarization (*P*_r_) can be used for non-volatile memory (NVM) application [8]. Material development for FeFETs recently has focused on polycrystalline-doped HfO_2_ due to its better thickness scalability [9] and CMOS process compatibility [2]. However, there still exists a fundamental limit for HfO_2_ thickness scaling to avoid undesired gate leakage current; this in turn limits the FinFET [2]. Inspired by the nanocrystal MOS and memory device concept [10, 11], an insulating dielectric (DE) layer with embedded ferroelectric (FE) nanocrystals is introduced in this work. The resulting new device design illustrated in Fig. 1 is called the “Nanocrystal-Embedded-Insulator” (NEI) FeFET. The main advantage of this design is a thinner unified-FE/DE layer that meets the low-gate-leakage requirement.

In this work, NEI FeFETs are reported. Physical properties and ferroelectricity of the NEI layers with different physical thicknesses are characterized. Electrical performance of NEI FeFETs is investigated for low-voltage logic and NVM applications.

## Methods

Key process steps for NEI FeFETs fabrication are shown in Fig. 1a. Four-inch n-type Ge(001) wafers with a resistivity of 0.088–0.14 Ω cm were used as the starting substrates. After pregate cleaning using diluted HF, Ge(001) wafers were loaded into an atomic layer deposition (ALD) chamber for the deposition of the NEI layer comprising ZrO_2_ nanocrystals embedded in amorphous Al_2_O_3_ matrix. NEI layers with the various thicknesses were utilized in this work. TaN metal gate was deposited on the NEI FeFETs using the reactive sputtering. After the gate patterning and etching, BF_2_^+^ ions were implanted into the source/drain regions at an energy of 20 keV and a dose of 1 × 15 cm^−2^. Thirty-nanometer nickel (Ni) was deposited in source/drain regions using the lift-off process. Finally, device fabrication was completed with rapid thermal annealing (RTA). Control metal-oxide-semiconductor field-effect transistors (MOSFETs) with a purely dielectric Al_2_O_3_ gate insulating layer also were fabricated.

Figure 1b shows the 3D schematic of the fabricated NEI FeFET, which comprises FE nanocrystals embedded in an amorphous DE gate insulating layer. Although the volume of FE material is small, it is sufficient for NCFET and NVM applications. The insulating DE material is key to achieving low gate leakage and low operating voltage; it should have both a large bandgap energy and high dielectric permittivity (*κ*). It also should provide for a high coercive field (*E*_c_) of the embedded FE nanocrystals.

**Fig. 2:**
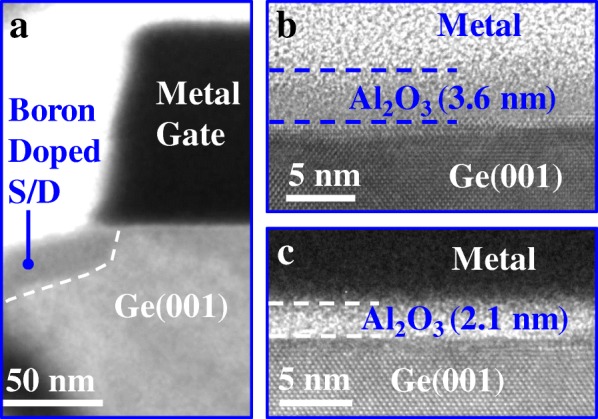
**a** XTEM image showing gate, channel, and source/drain regions of NEI-FeFET. **b** and **c** XTEM images of gate stack of FeFETs with 3.6- and 2.1-nm-thick NEI layers, respectively

The cross-sectional transmission electron microscope (XTEM) image in Fig. 2a shows the source/drain, channel, and gate edge regions of a fabricated FeFET. Figures 2b and c indicate the thicknesses of the NEI layers studied in this work to be 3.6 and 2.1 nm, respectively. Note that an interfacial layer of GeO_*x*_ exists between the NEI layer and Ge, although it cannot be seen.

**Fig. 3:**
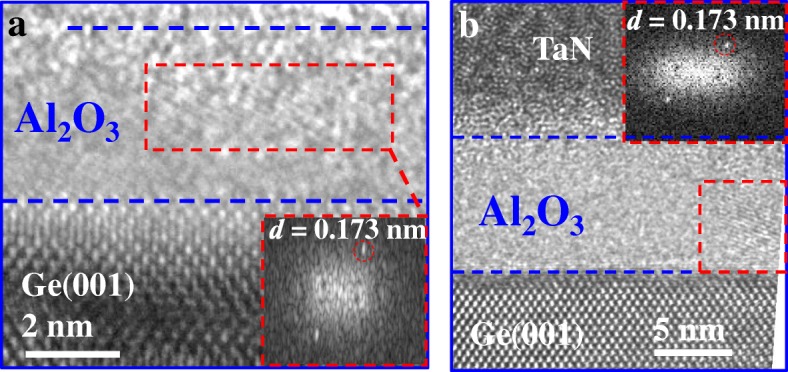
HRTEM images showing nanocrystals embedded in amorphous Al_2_O_3_ for the samples with thicknesses of **a** 3.6 nm and **b** 6 nm. Insets show that the interplanar spacing *d* in the nanocrystal is 0.173 nm, corresponding to o-ZrO_2_(111) phase

High-resolution TEM (HRTEM) images in Fig. 3 demonstrate the ZrO_2_ nanocrystals embedded in amorphous Al_2_O_3_ on Ge(001) in the NEI samples with thicknesses of 3.6 and 6 nm. In our previous work, we have shown that the atomic percentage of Zr in the NEI layer is less than 0.5% [12]. Based on the diffraction patterns, the interplanar spacing *d* within the nanocrystals is calculated to be 0.173 nm, which corresponds to (111)-oriented orthorhombic ZrO_2_ phase [13].

**Fig. 4:**
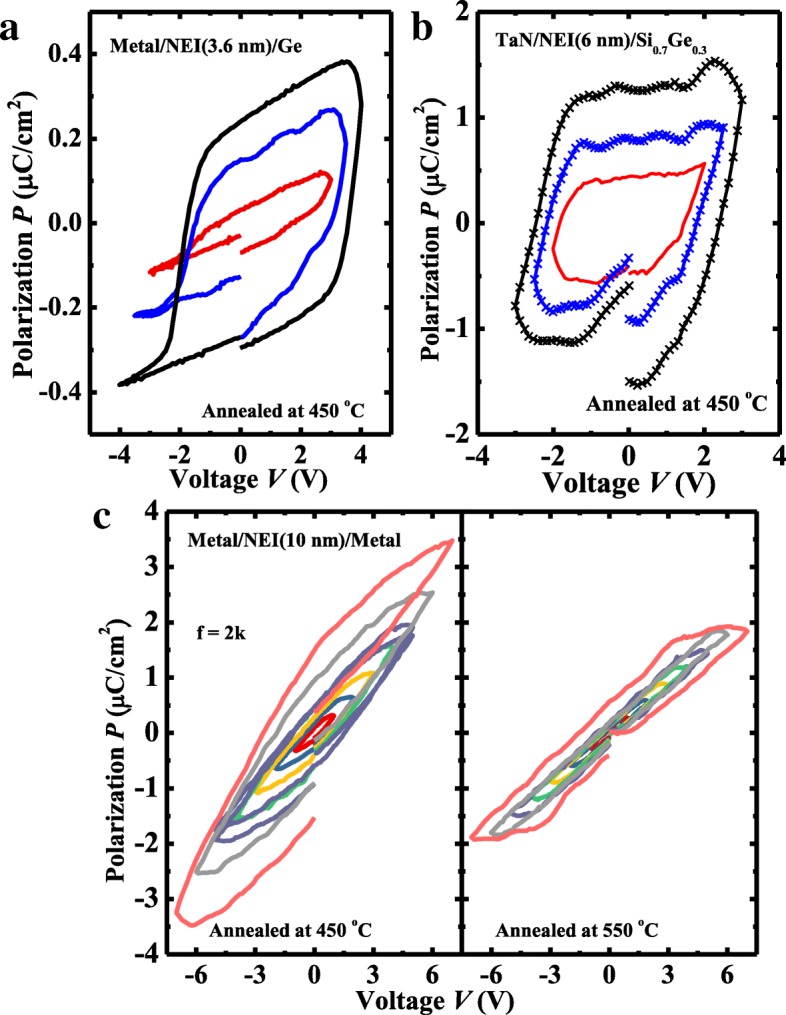
**a**–**c** Measured *P*-*V* curves of TaN/NEI (3.6 nm)/Ge, TaN/NEI (6 nm)/Si_0.7_Ge_0.3_, and TaN/NEI (10 nm)/TaN, respectively

**Fig. 5:**
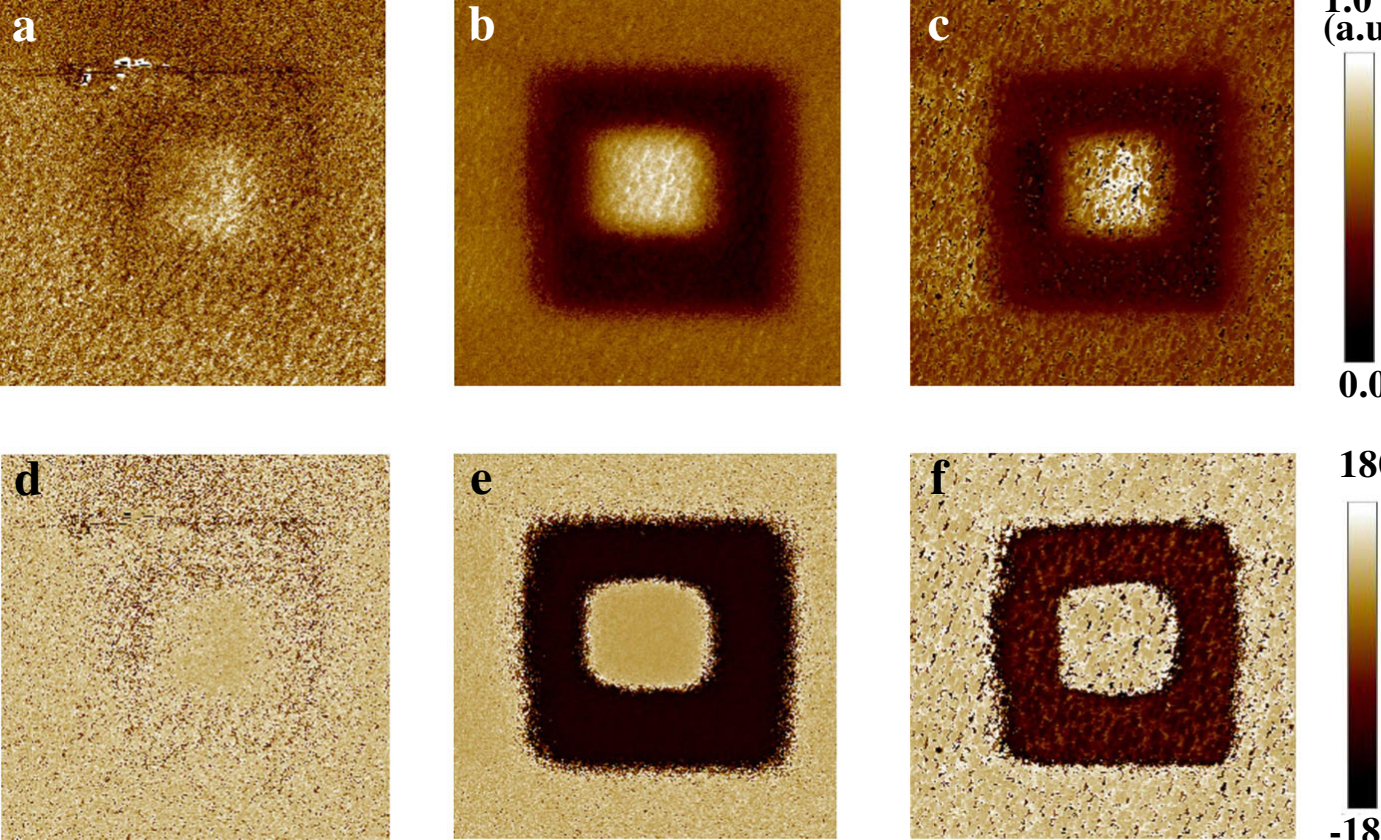
**a**–**c** Amplitude (upper) and phase (lower) images of PFM measurement for 3.6, 6, and 10 nm NEI on TaN, respectively

**Fig. 6:**
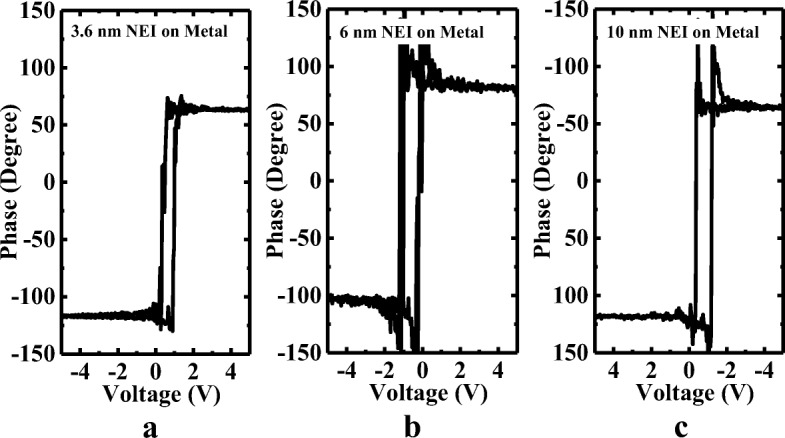
**a**–**c** Phase change characteristics of 3.6, 6, and 10 nm NEI on TaN, respectively. It is observed that opposite polarity can be written onto the surface of the NEI layer

Polarization vs. voltage (*P*-*V*) and piezoresponse force microscopy (PFM) measurements were carried out on the NEI samples with the different thicknesses. To characterize the ferroelectricity of the NEI layer, *P*-*V* curves of TaN/NEI (3.6 nm)/Ge, TaN/NEI (6 nm)/Si_0.7_Ge_0.3_, and TaN/NEI (10 nm)/TaN capacitors are shown in Fig. 4a, b, and c, respectively. The NEI layer exhibits a lower *P* than the reported values of HfZrO_2_ (HZO) [14], which is due to the fact that the volume ratio of ZrO_2_ nanocrystal in Al_2_O_3_ matrix is quite low. It is seen that the remnant polarization *P*_r_ of the NEI film increases with the increasing of film thickness. *P-V* curves in Fig. 4c indicate that the ferroelectricity of the NEI layer degenerates while the annealing temperature increases from 450 to 550 °C. It is noted that the reason for the unclosed *P-V* loops is because a leakage indeed exists. It was reported that the resultant offset at zero electric field diminishes as the voltage sweeping range is reduced [3, 15, 16]. The amplitude (upper) and phase (lower) images of 3.6 nm, 6 nm, and 10 nm NEI were measured, as shown in Fig. 5a, b, and c, respectively. As shown in Fig. 6, patterns indicating the opposite polarity written onto the surface of NEI on TaN exhibit the clearer contrast with the increasing of film thickness.

## Results and Discussion

### NEI NCFET

**Fig. 7:**
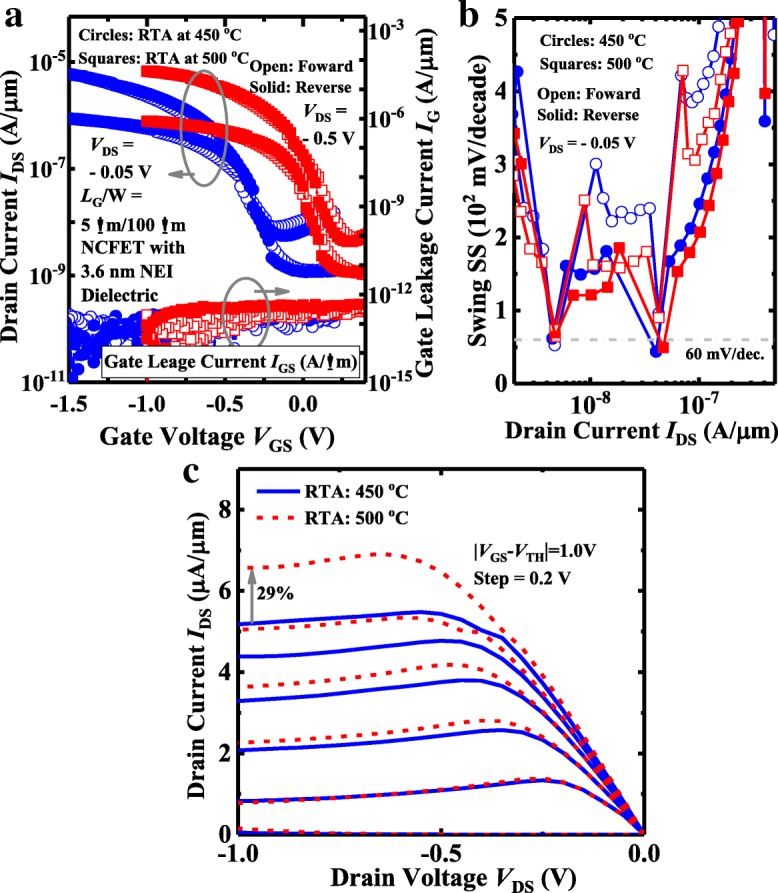
**a** Measured *I*_DS_-*V*_GS_ and *I*_G_-*V*_GS_ curves of NCFETs with 3.6-nm NEI annealed at 450 °C and 500 °C. **b** NEI NCFETs has the sub-60 mV/decade points for a *V*_DS_ value of − 0.05 V. **c** *I*_DS_-*V*_DS_ curves for the NEI NCFETs showing the obvious NDR phenomena. NC transistor annealed at 500 °C achieves a 29% *I*_DS_ improvement compared to the device with RTA at 450 °C at a supply voltage of 1.0 V

Figure 7a shows measured *I*_DS_-*V*_GS_ curves of the NEI NCFETs with a NEI thickness of 3.6 nm annealed at 450 °C and 500 °C. The NCFETs exhibit little hysteresis indicating the good matching between the ferroelectric capacitance and the MOS capacitance in the transistors. The NCFETs show the NC effect induced clockwise *I-V* loops, which is in contrast to the counterclockwise ones by charge trapping/detrapping [17]. The gate leakage *I*_G_ as a function of *V*_GS_ of the same pair of devices demonstrates that the formation of nanocrystals in Al_2_O_3_ does not increase the gate leakage. Figure 7b shows that the NCFETs achieve the sub-60 mV/decade steep SS points for the forward and reverse sweepings. The SS fluctuations in the NEI NCFET, also observed in NC FinFETs [2, 18], might be due to the polarization switching by the different ferroelectric nanocrystals or domains. The measured *I*_DS_-*V*_DS_ curves for the same pair of devices in Fig. 7c show that at ∣*V*_GS_ − *V*_TH_ ∣  =  ∣ *V*_DS_ ∣  = 1.0 V, the NCFET with RTA at 500 °C achieves 29% larger *I*_DS_ in comparison with the transistor annealed at 450 °C. This is attributed to the fact that the carrier mobility in channel and contact resistance characteristics can be improved with the increasing of annealing temperature [19]. The typical characteristic induced by the ferroelectric layer, negative differential resistance (NDR), is observed in the *I*_DS_-*V*_DS_ curves for the NCFETs annealed at the different temperatures.

**Fig. 8:**
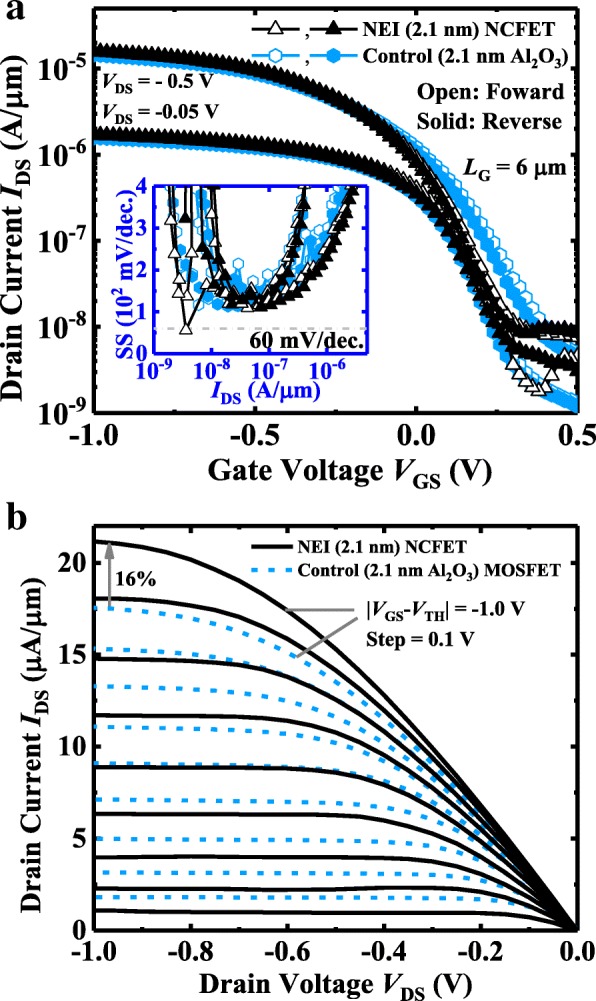
**a** *I*_DS_-*V*_GS_ curves of an NEI NCFET and control MOSFET with pure Al_2_O_3_ dielectric. Both devices have the 2.1-nm gate insulator. The inset shows that the NCFET has steeper SS than control device up to the threshold voltage. **b** Measured *I*_DS_-*V*_DS_ curves for NCFET and control MOSFET. NDR is observed for NCFET at very low *V*_GS_. At ∣*V*_GS_ − *V*_TH_ ∣  =  ∣ *V*_DS_ ∣  = 1.0 V, NCFET achieves a 16% *I*_DS_ improvement compared to the control device

Figure 8a shows measured *I*_DS_-*V*_GS_ curves of a NEI NCFET and a control MOSFET with the same insulator thickness of 2.1 nm. Devices have a *L*_G_ of 6 μm. The NCFET exhibits the hysteresis-free characteristics. The inset shows the point SS vs. *I*_DS_ curves for the devices, demonstrating that improved SS is achieved in the NCFET compared to the control device, up to the threshold voltage. Figure 8b shows the *I*_DS_-*V*_DS_ curves of the NEI NCFET and the control MOSFET. NCFET exhibits the NDR phenomenon for the low *V*_GS_. The NDR effect corresponds to the improved drain-induced barrier lowering (DIBL) characteristics in NCFET compared to the control MOSFET, as shown in Fig. 8a. At ∣*V*_GS_ − *V*_TH_ ∣  =  ∣ *V*_DS_ ∣  = 1.0 V, a 16% *I*_DS_ enhancement is obtained in NCFET in comparison with the control device. NCFET with 2.1 nm NEI has the less significant NDR compared to the transistor with 3.6 nm NEI, which is consistent with the conclusion in [20].

**Fig. 9:**
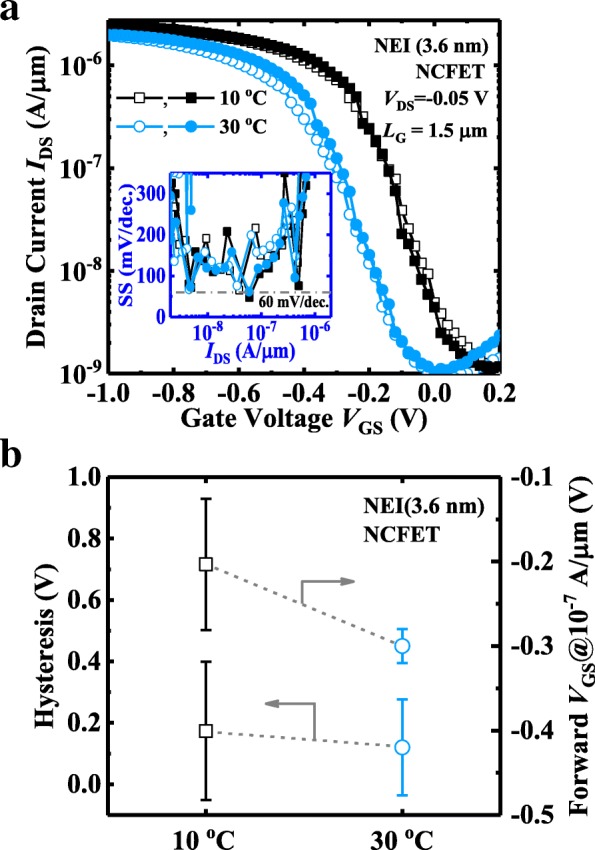
**a** *I*_DS_-*V*_GS_ of a NEI (3.6 nm) NCFET measured at 10 °C and 30 °C. The curves show a shift towards more negative voltage with increasing temperature, as expected. Inset shows steep point SS. **b** Statistical plots of hysteresis (left) and forward *V*_GS_ @ 10^−7^ A/μm (right) for NCFETs with 3.6-nm NEI layer. Forward *V*_GS_ shifts in the negative direction with increasing temperature

The temperature dependence of the NCFET with 3.6-nm-thick NEI is investigated herein. Figure 9a shows *I*_DS_-*V*_GS_ curves measured at 10 °C and 30 °C. Inset indicates that the SS performance of the transistor does not degrade at the elevated temperatures. As the temperature increases, the *I*-*V* curve shifts to more negative *V*_GS_ due to the dominant effect of ferroelectricity, which is opposite to the trend for a conventional MOSFET. Figure 9b summarizes the shifts in hysteresis voltage and forward switching threshold voltage with temperature. Forward *V*_GS_ shifts to more negative values as temperature increases, which might be due to increased *E*_c_ of the NEI.

### NEI FeFET for Non-Volatile Memory Application

**Fig. 10:**
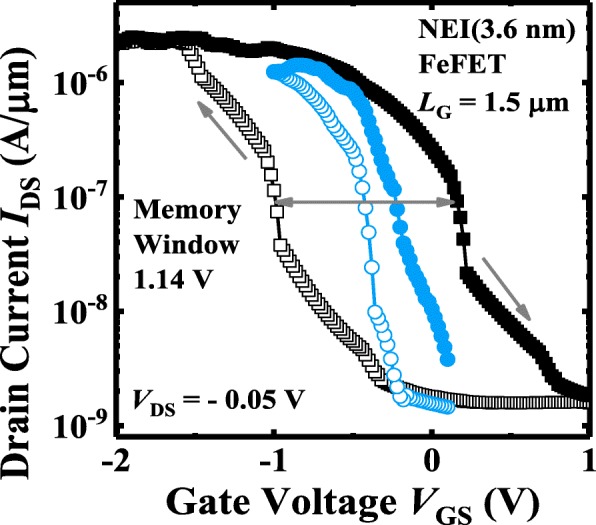
For a large *V*_GS_ DC sweeping range, a MW of 1.14 V is observed for the NEI (3.6 nm) FeFET

**Fig. 11:**
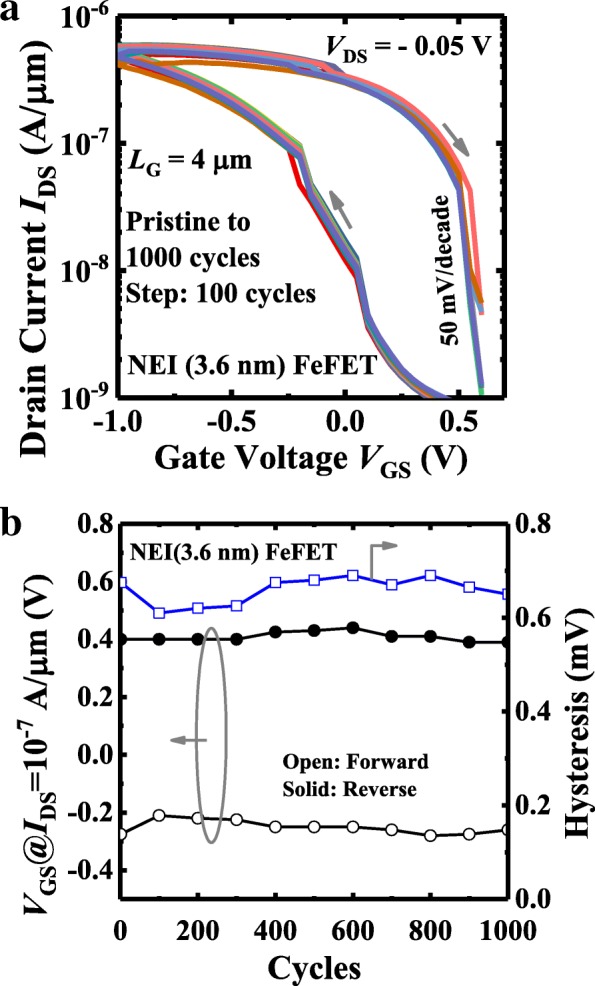
**a** Measured *I*_DS_-*V*_GS_ curves for NEI (3.6 nm) FeFET, through 1000 DC sweeping cycles. **b** DC sweeping endurance measurements show that the NEI FeFET has a stable MW through 1000 cycles

By increasing the range of *V*_GS_ sweeping, the hysteresis voltage of a NEI FeFET can be increased to achieve a large and stable memory window (MW) for read and write operations. As shown in Fig. 10, a FeFET with 3.6-nm NEI demonstrates that the MW increases from 0.2 to 1.14 V as *V*_GS_ sweeping range varies from (0.1 V, − 0.1 V) to (1 V, − 2 V). DC sweep endurance of another FeFET memory device is shown in Fig. 11a, Fig. 11b illustrates the hysteresis characteristics as a function of number of DC sweeping cycles. Stable *I-V* hysteresis window of ~ 0.65 V is seen.

**Fig. 12:**
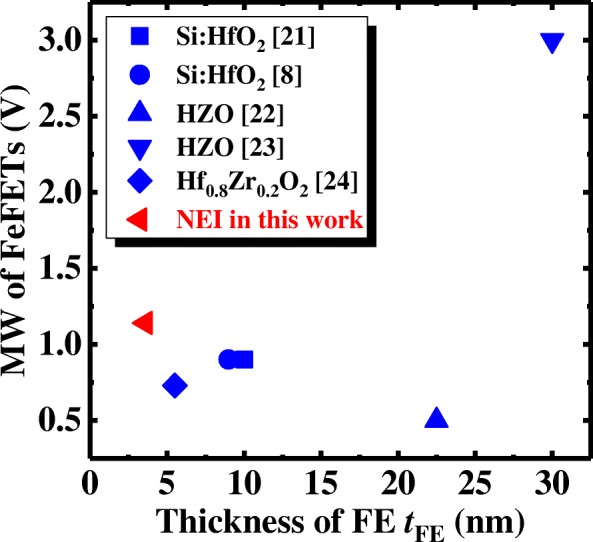
Benchmarking of NEI FeFET memory device against reported FeFETs, with regard to MW and *t*_FE_. Thinnest FE is achieved by NEI FeFET memory device

Figure 12 benchmarks the NEI FeFET memory device against reported FeFETs, with regard to MW and FE layer thickness [8, 21–24]. It should be noted that the NEI FeFET device in this work achieves a sizable (> 1 V) MW with the thinnest reported FE thickness of 3.6 nm. We speculate that it is easier to achieve the stable FE phase in NEI with a smaller thickness, as compared to the doped HfO_2_ [28–30].

**Fig. 13:**
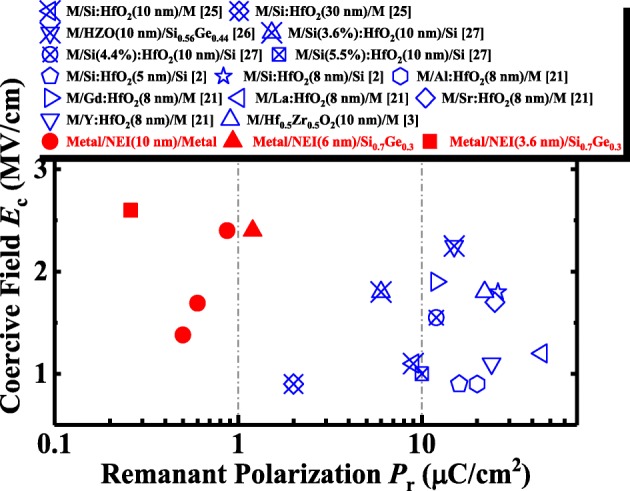
Benchmarking of NEI layers against reported doped HfO_2_ films, with regard to *E*_c_ and *P*_r_. NEI achieves much lower *P*_r_ compared to doped HfO_2_ while maintaining similar *E*_c._ [2, 3, 21, 25–27]

Finally, the advantages of the NEI FeFET provided by ZrO_2_ nanocrystals embedded in amorphous gate insulator are discussed. Figure 13 benchmarks the NEI layer against reported doped HfO_2_ films [2, 3, 21, 25–27], with regard to *E*_c_ and *P*_r_. NEI can achieve a much lower *P*_r_ compared to doped HfO_2_ for similar *E*_c_. Our experiments have demonstrated that a *P*_r_ below 1 μC/cm^2^ can provide the required MW in the FeFETs. Excessive polarization could lead to greater depolarization, resulting in worse retention characteristics, which was reported in [25]. Furthermore, the FE and DE properties of the NEI layer can be adjusted separately: *P*_r_ is enhanced/reduced by increasing/decreasing the volume of FE nanocrystals, and κ is increased by incorporating other elements in the amorphous matrix (e.g*.*, LaAlO_3_), to optimize FeFET performance.

## Conclusions

Novel FeFETs with ZrO_2_ nanocrystals embedded in an amorphous Al_2_O_3_ gate insulating layer are reported. Physical analyses indicate that less than 0.5% Zr in Al_2_O_3_ produces sufficient ferroelectricity for NCFET and NVM applications. Stable NC effect is observed at different measurement temperatures. Stable FeFET memory operation with record thin (3.6-nm total thickness) gate insulator is demonstrated. Stable MW is achieved over 1000 DC endurance cycles. The proposed NEI FeFET design provides a pathway for scaling down the thickness of the FE/DE gate insulator layer to be compatible with FinFETs with very small fin pitches.

## Abbreviations

Al_2_O_3_: Aluminum oxide
ALD: Atomic layer deposition
BF_2_^+^: Boron fluoride ion
DC: Direct current
Ec: Coercive field
FeFET: Ferroelectric field-effect transistor
Ge: Germanium
GeO_*x*_: Germanium oxide
HF: Hydrofluoric acid
HRTEM: High-resolution transmission electron microscope
*I*_DS_: Drain current
MOSFETs: Metal-oxide-semiconductor field-effect transistors
MW: Memory window
NC: Negative capacitance
NDR: Negative differential resistance
NEI: Nanocrystal-embedded-insulator
Ni: Nickel
Pr: Remnant polarization
RTA: Rapid thermal annealing
SS: Subthreshold swing
TaN: Tantalum nitride
*V*_GS_: Gate voltage
*V*_TH_: Threshold voltage
ZrO_2_: Zirconium dioxide
